# Urinary dysfunction in women following total mesorectal excision versus partial mesorectal excision for treatment of rectal cancer

**DOI:** 10.1186/s12905-021-01381-7

**Published:** 2021-06-07

**Authors:** Henry H. Chill, Shani Y. Parnasa, Noam Shussman, Roie Alter, Briggite Helou, Adiel Cohen, Alon J. Pikarsky, David Shveiky

**Affiliations:** 1 Division of Female Pelvic Medicine and Reconstructive Surgery, Department of Obstetrics and Gynecology, Hadassah Medical Organization and Faculty of Medicine, Hebrew University of Jerusalem, Jerusalem, Israel; 2 Department of Obstetrics and Gynecology, Hadassah Medical Organization and Faculty of Medicine, Hebrew University of Jerusalem, Jerusalem, Israel; 3 Department of Surgery, Hadassah Medical Organization and Faculty of Medicine, Hebrew University of Jerusalem, Jerusalem, Israel

**Keywords:** Rectal cancer, Total mesorectal excision, Partial mesorectal excision, Urinary dysfunction

## Abstract

**Background:**

Colorectal cancer is a condition which is associated with substantial morbidity and mortality. The aim of this study was to assess urinary dysfunction and its effect on quality of life in women who underwent total mesorectal excision compared to women treated by partial mesorectal excision for treatment of rectal cancer.

**Methods:**

We performed a retrospective cohort study at a tertiary university hospital between January 2014 and December 2019. A comparison was performed between women who underwent total mesorectal excision as opposed to partial mesorectal excision for treatment of rectal cancer. Pre-operative, intra-operative and post-operative data were compared between groups. Data regarding radiation therapy was recorded and compared as well. Urinary dysfunction and its impact on quality of life were assessed using UDI-6 and USIQ questionnaires. Further univariate and multivariate analyses were performed in the attempt of assessing risk factors for urinary dysfunction.

**Results:**

A total of 107 women were included in the study, 73 women underwent partial mesorectal excision as opposed to 34 women who were treated by total mesorectal excision. Twenty-five women in the TME group underwent radiation therapy prior to surgery as opposed to none in the PME group (*p* < 0.001). Urinary dysfunction following surgery as assessed using the UDI-6 questionnaire did not differ between groups. Similar findings were recorded with regard to the impact of urinary dysfunction on quality of life as assessed using the USIQ questionnaire. Following multivariate analysis longer hospital stay was associated with increased risk of some degree of urinary dysfunction.

**Conclusions:**

Women undergoing total mesorectal excision have comparable results to partial mesorectal excision with regard to urinary dysfunction.

## Background

Colorectal cancer (CRC) is a condition which is associated with substantial morbidity and mortality on a global scale. According to estimates it is the third most commonly diagnosed cancer and the fourth leading cause for cancer mortality worldwide [1–3]. A recent rise in five year survival rates has led to the need of addressing issues pertaining to quality of life and wellbeing of CRC survivors [4].

Surgical treatment is the hallmark of CRC management and includes several procedures most of which aim to achieve complete remission. While during resection of proximal tumors, advanced dissection of the pelvic region is avoided, more distal tumors, including mid to low rectal involvement, require a lower resection. This may compromise neurovascular structures in the surgical site resulting in impaired function of adjacent organs. Two of these procedures are total mesorectal excision (TME) and partial mesorectal excision (PME). Since introduced in 1982, TME has been considered the standard technique for treatment of rectal cancer showing favorable oncological outcomes at the cost of substantial morbidity [5–8]. Many of the patients require preoperative radiotherapy or chemo-radiotherapy due to locally advanced disease, which can worsen functional outcomes [9]. More recent data has pointed towards the possibility of utilizing a less radical approach, that is PME, according to tumor location. Advantages of this technique include achieving comparable oncological outcomes with decreased morbidity [10, 11].

Following surgical treatment for rectal cancer, women are at increased risk for pelvic floor dysfunction (PFD) including bowel, urinary and sexual disturbances [12, 13]. However, data comparing the effect of different surgical procedures on PFD is scarce and studies focusing on women in this clinical scenario are limited.

The aim of this study was to assess urinary dysfunction and its effect on quality of life in women diagnosed with rectal cancer who underwent TME for mid to low rectal tumors, compared to women treated by PME for upper rectal or distal sigmoid tumors.

## Methods

We performed a retrospective cohort study at a tertiary university teaching hospital between January 2014 and December 2019. Included were all women diagnosed with CRC, surgically treated during the study period and for whom surgical treatment included partial or complete resection of the rectum. Excluded were cases in which colectomy was performed due to benign indication, surgery which did not include any part of the rectum and women unable to answer telephone questionnaires. Informed consent was obtained from all women participating in the study. Institutional ethical review board approval was received (0413-19-HMO).

Women included were divided into two groups according to surgical procedure. The first group included women who underwent segmental resection of the rectum including PME. In this group anastomosis was performed 6–15 cm above the dentate line. The second group included women with CRC who underwent TME in which anastomosis was equal to or under 5 cm from the dentate line. Radiation therapy was performed prior to surgery according to standard protocol for treatment of locally advanced mid to low rectal cancer. Radiation therapy protocol included 28 daily doses of 1.8 Gray per treatment over a period of 5 1/2 weeks combined with chemotherapy of Fluorouracil (5FU) meant to increase sensitivity to radiation treatment. Quality of pathological specimens was evaluated according to Quirke's classification of rectal cancer resection specimens [14].

Demographic, general medical history pre-operative, intra-operative and post-operative data were retrieved from electronic medical records. Information collected included age, Body Mass Index (BMI), smoking status, parity, comorbidities, radiotherapy before surgery, length of surgery, tumor size, complications, level of anastomosis, adjuvant treatment and length of hospital stay. Women were contacted via telephone and were requested to take part in the study. Following receipt of informed consent, they were asked to answer several questionnaires. Patients were asked to answer regarding urinary symptoms at present time. Urinary Distress Inventory Short Form (UDI-6) questionnaire was used to evaluate urinary dysfunction while the Urgency, Severity and Impact (USIQ) questionnaire aimed to estimate effect of urinary dysfunction on quality of life (QOL). The UDI-6 questionnaire includes 6 items focusing on irritative, stress and obstructive symptoms. The USIQ questionnaire includes an initial filter questions followed by 5 questions regarding urgency symptoms and severity. The second part of the questionnaire consists of 8 questions focusing on the impact urinary symptoms have on QOL. For both questionnaires higher score indicates more impaired urinary function. All questionnaires have previously been validated to the Hebrew language [15].

The primary outcome of the study was difference in urinary dysfunction between the groups, as assessed by the UDI-6 questionnaire score. Secondary outcomes included difference in impact of urinary disorders on quality of life, assessed by (USIQ) questionnaire, and assessment of other risk factors associated with urinary disorders.

### Statistical analysis

The statistical software package SPSS 24.0 (SPSS Inc., Chicago, IL) was used for all data analyses. The chi-square and Fischer exact tests were used for categorical variables and the t-test and Mann–Whitney tests for continuous variables- all distributions were different from normal. Logistic regression was used for multivariate analysis, adjusting for available baseline, intra-operative and surgical characteristics. We report odds ratios (OR), 95% confidence interval (CI) for parameters included in the final multivariate analysis. A two-sided *p* values, with a value of < 0.05 were considered significant.

### Informed consent

Informed consent was obtained during telephone questionnaire from all women taking part in the study.

## Results

Following implementation of our exclusion criteria a total of 107 women were included in the study, 73 women underwent PME (PME group) as opposed to 34 women who were treated by TME (TME group). Surgery was performed laparoscopically for all patients except for one case in which conversion to open surgery was performed due to technical difficulty. A comparison of basic and pre-operative characteristics is presented in Table 1. Mean age was 60.7 (SD = 9.5) and 61.2 (SD = 12.6) in the PME and TME groups, respectively (*p* = 0.813). Women in the TME group had higher preoperative ASA score compared to the PME group. Twenty-five women in the TME group underwent radiation therapy prior to surgery as opposed to none in the PME group (*p* < 0.001). Other parameters assessed such as BMI, smoking status and other comorbidities did not differ between the groups.

**Table 1:** Basic and pre-operatvie characteristics of the study population—PME versus TME

Parameter	PME (n = 73)	TME (n = 34)	*p* value
Age	60.7 ± 9.5	61.2 ± 12.6	0.813
Smoker	5 (7.1%)	2 (6.3%)	0.907
Never	58 (82.9%)	28 (87.5%)	
Past	7 (10.0%)	2 (6.3%)	
Current	5 (7.1%)	2 (6.3%)	
BMI	27.7 ± 5.8	28.1 ± 4.7	0.785
Parity	3.6 ± 2.4	4.7 ± 2.9	0.179
Comorbidities			
Hypertension	6 (8.2%)	1 (2.9%)	0.304
Dyslipidemia	16 (21.9%)	10 (29.4%)	0.400
DM	10 (13.7%)	4 (11.8%)	1.000
IHD	3 (4.1%)	0 (0.0%)	0.550
AF	3 (4.1%)	1 (2.9%)	1.000
ASA			
0	0 (0.0%)	4 (11.8)	0.028
1	26 (35.6%)	10 (29.4%)	
2	41 (56.2%)	19 (52.9%)	
3	3 (8.2%)	1 (2.9%)	
Radiation before surgery	0 (0.0%)	25 (73.5%)	< 0.001

Data presented as mean ± SD or n (%)

*PME* partial mesorectal resection, *TME* total mesorectal resection, *BMI* body mass index, *DM* diabetes mellitus, *IHD* ischemic heart disease, *AF* atrial fibrillation, *ASA* American Society of anesthesiologists physical status classification system

Intra-operative and post-operative data as well as questionnaire scores are presented in Table 2. Mean length of surgery and complication rate were similar between groups. Women in the PME group had larger tumor size (4.0 ± 1.9 vs. 2.6 ± 1.2 cm, *p* < 0.001) and were discharged earlier from the hospital (mean hospital stay 7.6 ± 2.8 vs. 9.2 ± 3.2 days, *p* = 0.013) compared to women in the TME group. No difference was found with respect to need of adjuvant therapy between groups. All patients were negative for distal and circumferential resection margins.

**Table 2:** Intra-operative data and post-operative outcomes—PME versus TME

Parameter	PME (n = 73)	TME (n = 34)	*p* value
Length of surgery (minutes)	191 ± 83	229 ± 115	0.054
Intra-operative complications			
Hemorrhage	1 (1.4%)	2 (5.9%)	0.237
Unplanned stoma	0 (0.0%)	1 (2.9%)	0.318
Tumor size (cm)	4.0 ± 1.9	2.6 ± 1.2	< 0.001
Level of anastomosis from AV (cm)	13.4 ± 5.0	3.5 ± 2.3	< 0.001
Adjuvant treatment			0.783
None	34 (47.2%)	18 (52.9%)	
Chemotherapy	37 (51.4%)	16 (47.1%)	
Radiotherapy	1 (1.4%)	0 (0.0%)	
Hospital stay (days)	7.6 ± 2.8	9.2 ± 3.2	0.013
Post-operative complications			
Ileus	1 (1.4%)	1 (2.9%)	0.537
Infection	5 (6.8%)	1 (2.9%)	0.662
Bleeding	2 (2.7%)	1 (2.9%)	1.000
Leakage	0 (0%)	1 (2.9%)	0.318
High output stoma	0 (0%)	5 (14.7%)	0.003
Follow up (months)	34.3 ± 21.2	22.6 ± 18.6	0.007
UDI-6 score	11.6 ± 19.5	10.2 ± 16.9	0.989
USIQ score	11.0 ± 20.9	11.3 ± 20.3	0.512
Questions 1–5	14.0 ± 25.0	16.0 ± 25.6	0.422
Questions 6–13	9.0 ± 19.3	8.6 ± 20.7	0.938

Data presented as mean ± SD or n(%)

*PME* partial mesorectal resection, *TME* total mesorectal resection, *AV* anal verge, *UDI-6* urinary distress inventory short form, *USIQ* Urgency Severity and Life Impact Questionnaire

Urinary dysfunction following surgery as assessed using the UDI-6 questionnaire did not differ between groups (11.6 ± 19.5 vs. 10.2 ± 16.9, *p* = 0.989, for PME and TME groups, respectively). Similar findings were recorded upon comparison of the impact of urinary dysfunction on quality of life (QOL) as assessed using the USIQ questionnaire (Table 2).

Subgroup analyses were performed comparing TME and PME groups focusing on women with any urinary dysfunction (UDI-6 > 0) as well as women with more severe urinary dysfunction (UDI-6 > 25). Proportion of women with any urinary dysfunction did not differ between groups (28.8% vs. 35.3%, *p* = 0.496, for PME and TME groups, respectively). Similar findings were noted with respect to more severe cases of urinary dysfunction (15.1% vs. 17.6%, *p* = 0.734, for PME and TME groups, respectively).

We further analyzed the effect of time elapsed from surgery on urinary dysfunction. Women who underwent surgery within the previous year had similar UDI-6 scores compared to women with a time interval since surgery of over one year (14.5 ± 21.3 vs. 9.8 ± 17.3, *p* = 0.23, for < 1 year and > 1 year, respectively).

Univariate and multivariate analyses were performed, in the attempt of detecting risk factors associated with any urinary dysfunction within our cohort. Results of the stepwise logistic regression model can be found in Table 3. The logistic regression model was implemented for women with any level of urinary dysfunction (UDI-6 > 0), women with more severe urinary dysfunction (UDI-6 > 25) and for women with any impact of urinary dysfunction on quality of life (USIQ > 0). Following multivariate analysis women with longer hospitalization (OR = 1.38, CI 1.1–1.72, *p* = 0.005) had increased risk of having some level of urinary dysfunction. Regarding impact of urinary dysfunction on quality of life, increased hospital stay was associated with a detrimental effect on quality of life (OR = 1.36, CI 1.0–1.84, *p* = 0.05). Higher level of anastomosis (OR = 0.90, CI 0.83–0.98, *p* = 0.013) and absence of comorbidities decreased the risk of urinary dysfunction (OR = 0.19, CI 0.05–0.81, *p* = 0.025).

**Table 3:** Multivariate analysis of parameters associated with any and severe urinary dysfunction

	OR	95% CI	*p* value
UDI-6 > 0			
Increased hospital stay	1.38	1.1–1.72	0.005
No comorbidities	0.40	0.13–1.21	0.105
1 year < since surgery	2.52	0.79–8.06	0.118
UDI-6 > 25			
No comorbidities	0.27	0.07–1.11	0.069
Level of anastomosis	0.92	0.83–1.03	0.143
USIQ > 0			
Increased hospital stay	1.36	1.00–1.84	0.050
No comorbidities	0.19	0.05–0.81	0.025
Level of anastomosis	0.90	0.83–0.98	0.013
1 year < since surgery	2.72	0.60–12.35	0.194

*UDI-6* urinary distress inventory short form, *USIQ* Urgency Severity and Life Impact Questionnaire

## Discussion

In this study we assessed pelvic floor dysfunction in women following surgical treatment for rectal cancer. We found no difference, with respect to urinary dysfunction between women who underwent PME and those treated by TME. Similar findings were noted following comparison of the effect of urinary dysfunction on quality of life, between the two groups.

Few studies have focused on the effect of surgical treatment for rectal cancer on pelvic floor dysfunction in women. Daniels et al. contacted women following TME and evaluated urinary dysfunction [16]. They found nocturia and stress incontinence following surgery in 59% and 18% of women, respectively. Symptoms were predominant in women with low rectal cancers. Small sample size (n = 18) and lack of control group limit generalizability of these findings. In another study Böhm et al. reported on women who underwent TME and compared them to a control group of women who underwent colonic resection. They found urinary function to be normal in both groups while higher rate of anal incontinence was noted in the TME group [17].

During normal function, sympathetic nerves are responsible for inhibition of detrusor contraction as well as promotion of bladder neck constriction ensuring urinary continence. In contrast, the parasympathetic nerves innervate the detrusor and are responsible for muscle contraction essential for micturition. Furthermore, proprioceptive afferent fibers originating from the bladder wall have a key role in sensation of bladder filling and follow the same pathway as parasympathetic nerves [18, 19].

In theory, women undergoing TME could sustain damage to the hypogastric plexus leading to loss of sympathetic innervation, possibly causing urgency and stress incontinence. Moreover, damage to the splanchnic nerves could evoke detrusor denervation and desensitization with clinical manifestations of disturbance in bladder emptying and overflow incontinence. In our study we did not find increased risk of such disturbances in women undergoing TME compared to PME. This is an important finding since it may assist clinicians in the counseling of women who are candidates for surgical treatment of rectal cancer.

Most women in the TME group underwent radiation therapy prior to surgery as opposed to the PME group in which no women received such treatment. Several studies have shown increased urinary dysfunction in women treated with radiotherapy for colorectal malignancies compared to women treated by surgery alone [9, 20]. The fact that even following radiotherapy similar results were found between groups strengthens our findings that women treated by TME do not have increased urinary adverse events following surgery.

Following logistic regression, we found increased hospital stay to be an independent risk factor for any urinary symptoms. We did not however, find an effect of time elapsed from surgery on urinary dysfunction. Little is known to date, regarding the effect of time on urinary dysfunction in women following rectal cancer. Future studies with a larger cohort may assist in addressing this important question.

Strengths of the study include its comparative design and it being one of the only studies in women, comparing surgical approaches for treatment of colorectal cancer, with respect to pelvic floor dysfunction. Evaluation of pelvic floor dysfunction was achieved using both symptom and QOL validated questionnaires.

Limitations of the study include lack of baseline data regarding urinary dysfunction before surgery. No physical exam was performed which could have given valuable data pertaining to pelvic organ prolapse as well as clinical evaluation of stress urinary incontinence. Lastly, the study group was not large enough to assess risk factors for severe urinary dysfunction.

## Conclusion

In this study we show comparable outcomes between women treated by TME compared to PME, with respect to urinary dysfunction. These findings pertain to symptoms as well as their impact on QOL. Further studies are needed in order to investigate urinary dysfunction in colorectal cancer survivors but we believe these results may encourage clinicians to offer women optimal oncological treatment without compromising urinary function.

## Data Availability

The datasets used and/or analyzed during the current study are available from the corresponding author on reasonable request.

## Abbreviations

CRC: colorectal cancer
TME: total mesorectal excision
PME: partial mesorectal excision
PFD: pelvic floor dysfunction
UDI-6: urinary distress inventory short form
USIQ: urgency, severity and impact
QOL: quality of life
